# Ferroptosis boosting system based on a sonodynamic therapy cascade-augmented strategy for triple-negative breast cancer therapy

**DOI:** 10.1093/rb/rbaf042

**Published:** 2025-05-20

**Authors:** Juying Zhang, Hanmei Li, Litao Ye, Yihan Leng, Xiaoqing Wang, You Yang, Qiong Jiang, Linli Feng, Ling Li, Yang Li, Jinhong Yu

**Affiliations:** School of Medical Imaging, North Sichuan Medical College, Nanchong, Sichuan 637000, China; Innovation Centre for Science and Technology of North Sichuan Medical College, Nanchong, Sichuan 637000, China; School of Medical Imaging, North Sichuan Medical College, Nanchong, Sichuan 637000, China; Innovation Centre for Science and Technology of North Sichuan Medical College, Nanchong, Sichuan 637000, China; School of Medical Imaging, North Sichuan Medical College, Nanchong, Sichuan 637000, China; Innovation Centre for Science and Technology of North Sichuan Medical College, Nanchong, Sichuan 637000, China; School of Medical Imaging, North Sichuan Medical College, Nanchong, Sichuan 637000, China; School of Medical Imaging, North Sichuan Medical College, Nanchong, Sichuan 637000, China; School of Medical Imaging, North Sichuan Medical College, Nanchong, Sichuan 637000, China; School of Medical Imaging, North Sichuan Medical College, Nanchong, Sichuan 637000, China; School of Medical Imaging, North Sichuan Medical College, Nanchong, Sichuan 637000, China; School of Medical Imaging, North Sichuan Medical College, Nanchong, Sichuan 637000, China; School of Medical Imaging, North Sichuan Medical College, Nanchong, Sichuan 637000, China; School of Medical Imaging, North Sichuan Medical College, Nanchong, Sichuan 637000, China

**Keywords:** ferroptosis boosting system, reactive oxygen species, sonodynamic therapy, autophagy, cascade-augmented strategy

## Abstract

One of the novel forms of programmed cell death, ferroptosis, has recently emerged as a hopeful treatment strategy for triple-negative breast cancer (TNBC). However, insufficient levels of intracellular reactive oxygen species (ROS) and high levels of ROS scavengers in the tumor microenvironment (TME), such as glutathione (GSH), hamper the efficacy of ferroptosis therapy. In this study, the introduction of manganese dioxide nanoparticles (MnO_2_ NPs) generated cytotoxic hydroxyl radicals (⋅OH) in the TME. Importantly, MnO_2_ NPs act as a nanosensitizer by consuming H_2_O_2_/GSH in the TME, generating oxygen (O_2_) to relieve the oxygen deficiency of tumors, induce tumor oxidative stress and ultimately enhance SDT-induced ferroptosis. Additionally, oxygen, as an ultrasound contrast agent, enables the visualization of the TNBC treatment process. Meanwhile, GSH depletion in the TME leads to failure of the major cellular system defending against ferroptosis, which also promotes the accumulation of lipid peroxidation in tumor tissue. Specifically, robust autophagy induced by ROS enhances the intracellular iron pool by breaking down ferritin, thereby promoting ferroptosis in cancer cells, leading to the optimal antitumor effect. Consequently, a ferroptosis boosting system that simultaneously encapsulates MnO_2_ NPs and chlorin e6 (Ce6) was constructed for the intervention of TNBC. Both the *in vitro* and *in vivo* results demonstrated that Ce6-MnO_2_-BSA nanoparticles can generate a significant ROS storm under ultrasound irradiation, eliminating GSH and inducing an autophagic response that increases the effectiveness of ferroptosis, thus, inhibiting the growth of TNBC without obvious toxic side effects. This effective strategy can cascade-augment cancer cell ferroptosis, providing a new perspective for the clinical treatment of TNBC.

## Graphical abstract

**Figure:**
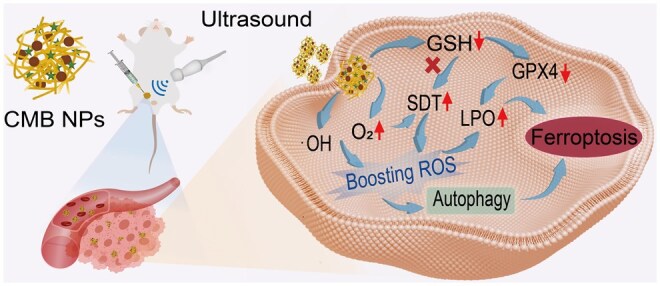

## Introduction

As reported by the Global Cancer Statistics 2022 of the World Health Organization, breast cancer is the most common type of cancer among women [1]. In particular, TNBC is one of the more critical subtypes of breast cancer due to its refractory nature, tendency to relapse and low survival rate [2]. However, the treatment methods for TNBC are complex and include surgery, chemotherapy, radiotherapy, immunotherapy and targeted therapy [3]. With a deeper understanding of cancer therapy mechanisms, some types of nonapoptotic cell death, like ferroptosis, autophagy, pyroptosis and cuproptosis, have been reported [4], providing new pathways for the management of TNBC.

Ferroptosis is a type of iron-dependent, nonapoptotic programmed cell death, distinguished by an increase in reactive oxygen species (ROS) and lipid peroxidation (LPO) that reach a lethal threshold, is widely used in TNBC treatment [5–8]. As a critical stage in the occurrence of ferroptosis, LPO depends on the generation of ROS/O_2_. Briefly, Lysophosphatidyl hydroperoxide (LH) is induced by ⋅OH first, which trigger the creation of lipid alkyl radicals (L). Subsequently, L⋅ combines with O_2_ to form lipid peroxides (LOO⋅). Then, the LOO⋅ abstracts a hydrogen atom (H) from another molecule of LH, thereby facilitating the release of lipid hydroperoxide (LOOH) as a result of this proton transfer process [9–13]. In addition, the SLC7A11–GSH–GPX4 axis plays a critical role as an essential component of the ferroptosis counteracting system, significantly contributing to the progression of ferroptosis [14]. During this process, glutathione (GSH) serves as a key electron donor, maintaining high levels within the cell. The overexpression of GSH facilitates the preservation of cellular redox homeostasis, thereby protecting cells from external oxidative stress-induced damage [15–17]. However, the efficiency of ferroptosis is severely impaired by the hypoxic nature and the overexpressed GSH (≈10 × 10^−3^ M) of the TME [18]. Therefore, increasing the generation of ROS/O_2_ and the consumption of GSH at the tumor site can enhance the efficacy of ferroptosis.

Sonodynamic therapy (SDT) has attracted significant interest because of its ability to penetrate tissues deeply and non-invasive characteristics [19–23]. Generally, under ultrasound (US) irradiation, the sonosensitizer Ce6 transfers energy to convert O_2_ into singlet oxygen (^1^O_2_), which induces LPO and results in irreversible ferroptosis [24, 25]. However, the typical O_2_ deficiency in the TME severely limits the therapeutic efficiency of SDT in the local treatment range [26]. Therefore, increasing the production of O_2_ at the tumor site is one of the most common strategies to enhance the efficacy of SDT (broaden sources). However, the overexpression of GSH in the TME can dramatically consume ROS generated by SDT, resulting in the ineffective inhibition of tumor growth [27]. GSH consumption provides an important idea for improving the efficacy of SDT (reduce expenditure). Thus, it is a potential strategy to optimize the efficacy of SDT by increase the production of O_2_ and the consumption of GSH at the tumor site.

MnO_2_ nanoparticles have been explored for cancer treatment to modulate intracellular ROS by generating ⋅OH through Mn^2+^-mediated Fenton-like reactions in slightly acidic TME [28]. Moreover, the storm of ROS upon ultrasound irradiation is attributed to the prominent increase in O_2_ levels and the consumption of GSH at the tumor site by MnO_2_. In addition, the depletion of GSH is an important factor leading to the inactivation of glutathione peroxidase 4 (GPX4, a ferroptosis inhibition protein), which significantly decreases the scavenging of LPO, inducing cancer cell ferroptosis [29, 30]. Hence, increasing the production of ROS triggered by SDT at the tumor site and the depletion of GSH, along with the exacerbation of LPO accumulation, are key features that make MnO_2_ an ideal candidate as a ferroptosis boosting system.

Among the various therapeutic mechanisms in cancer, in addition to ferroptosis, autophagy also plays an indispensable role. Autophagy is an essential lysosome-mediated degradation process that removes damaged organelles and misfolded proteins, making it a key strategy in future cancer therapies [31–35]. Increasing evidence suggests that excessive ROS can trigger a potent autophagic response, which has been demonstrated to augment cancer cell ferroptosis by degrading the intracellular iron storage protein ferritin [36–42]. Therefore, achieving triple-enhanced ferroptosis induced by SDT-induced ROS accumulation, GSH consumption and autophagy is of great significance for the clinical treatment of TNBC.

To address the current poor efficacy of treatment for TNBC, a rapid, simple and efficient method was used to reduce KMnO_4_ with the amine and thiol groups of biocompatible bovine serum albumin (BSA), resulting in a multifunctional nanoplatform based on BSA-templated MnO_2_ NPs. To load the sonosensitizer Ce6, the carboxyl group of Ce6 is activated using EDC to form an activated intermediate. Subsequently, to improve the reaction selectivity and reduce side products, NHS can react with EDC to form a more stable NHS ester intermediate, which can then react with an amino group (–NH_2_) to form an amide bond, while minimizing the production of undesirable by-products. When the Ce6-MnO_2_-BSA nanoparticles (designated CMB NPs) accumulate at the tumor site, the CMB NPs efficiently respond to the specific TME, triggering the generation of large quantities of ROS. Moreover, the production of O_2_ and the consumption of GSH by MnO_2_ can improve the production of ROS under ultrasonic irradiation of Ce6, which synergistically increases the accumulation of LPO. Most importantly, depleting GSH can inactivate the SLC7A11–GSH–GPX4 axis, which is a vital cellular system against ferroptosis, increasing the likelihood of being affected by tumor cells to ferroptosis. Finally, a high level of ROS can trigger a potent autophagic response that causes ferritin engulfment and degradation to amplify ferroptosis. Overall, this work provides a novel path to cascade-enhanced ferroptosis, significantly improving the therapeutic efficacy of TNBC treatment.

## Results and discussion

### Preparation and characterization of CMB NPs

**Figure 1.**
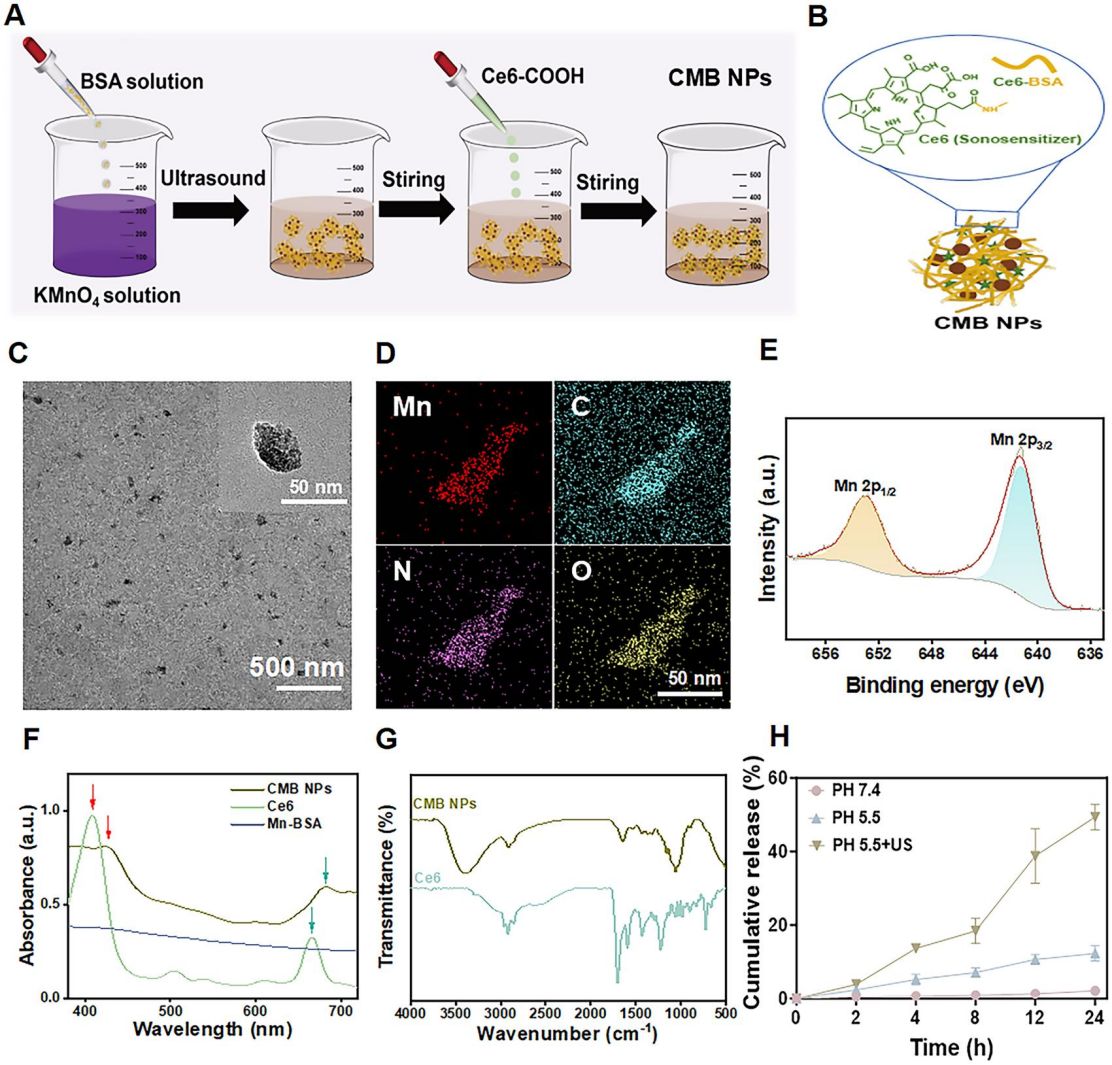
Preparation and characterization of CMB NPs. (**A**) Synthesis of CMB NPs. (**B**) The connection between Ce6 and BSA. (**C**) CMB NPs were observed by TEM. (**D**) The corresponding element mappings of Mn, C, N and O in CMB NPs. (**E**) X-ray photoelectron spectroscopy (XPS) spectra of Mn 2p. (**F**) UV–Vis spectroscopy of Ce6, Mn-BSA and CMB NPs. (**G**) FTIR spectra of CMB NPs, and Ce6. (**H**) The drug release from CMB NPs under different conditions.

Considerable interest has been attracted to BSA as a component of drug delivery systems due to its nontoxicity, low cost and good biocompatibility with the immune system [43, 44]. Importantly, BSA contains amino acid residues with reductive properties; therefore, we used BSA as both a carrier and reducing agent. In this process, KMnO_4_ was reduced to MnO_2_ through the active groups of BSA, creating conditions for the loading of MnO_2_ onto BSA, which significantly simplified the preparation process (Figure 1A). The loading of Ce6 was probably due to the amide reaction between BSA and Ce6-COOH (Figure 1B). The general morphology and particle size distribution of the prepared CMB NPs are shown in the TEM image (Figure 1C). The results exhibited an irregular aggregation pattern, with a particle size of about 40 nm. The DLS results were consistent with the TEM observations, indicating that the CMB NPs had good dispersibility, with an average hydrodynamic diameter of approximately 43.8 nm. When the size of the nanoparticles is less than 100 nm, tumor tissues increase the passive accumulation of nanoparticles to the tumor through the enhanced permeability and retention (EPR) effect, thereby boosting the retention of nanoparticles at the tumor site [45]. Moreover, the dynamic size distribution of the CMB NPs was relatively uniform, and the polymer dispersity index was 0.791 (Supplementary Figure S1). The zeta potential of the CMB NPs exhibited a negative value, approximately −33.3 mV (Supplementary Figure S2). Moreover, when the CMB NPs were placed in PBS containing 10% serum, the particle size of the CMB NPs was measured at 0, 1, 2, 3, 4, 5, 6 and 7 d. The results demonstrated that the particle size remained almost unchanged within 7 days, confirming that the CMB NPs exhibited good stability in the serum-containing peripheral environment (Supplementary Figure S3). The energy-dispersive X-ray spectroscope (EDS) mapping of the CMB NPs was corresponding to the Figure 1D. The distribution of Mn, C, N and O was present in CMB NPs, indicating the successful doping of the Mn element. The composition analysis of CMB NPs was performed by X-ray photoelectron spectra (XPS). The XPS spectra of CMB NPs showed the characteristic double peaks at 652.9 eV and 641.2 eV that corresponded to Mn 2p½ and Mn 2p3/2, respectively, indicating that the Mn in CMB NPs was mainly in the form of valence IV (Figure 1E and Supplementary Figure S4). The above results showed that we successfully coated the surface of CMB NPs with MnO_2_.

The successful loading of the sonosensitizer Ce6 onto the CMB NPs was detected by UV–Vis spectroscopy (Figure 1F) and Fourier-transform infrared (FTIR) spectra (Figure 1G), respectively. No characteristic absorption peak similar to that of Ce6 was observed in the UV absorption spectrum of Mn-BSA. However, two absorption peaks, resembling the characteristic peaks of Ce6, were observed in the UV absorption spectrum of the CMB NPs (as indicated by the red and blue arrows in the figure). These results suggested that Ce6 was successfully loaded onto the CMB NPs. Moreover, the drug loading of Ce6 reached 56.8%, as calculated by UV–Vis spectroscopy. Furthermore, the FTIR spectra showed that the absorption peak at 3400 cm^−1^ was the N − H stretching vibration peak of CMB NPs and 1647 cm^−1^ was the C = O stretching vibration peak. All the above results indicated that Ce6 has been successfully loaded. Subsequently, the release efficiency of Ce6 under different conditions was investigated. As shown in Figure 1H, at a pH of 7.4 in PBS solution, the cumulative release rate of Ce6 was only about 2.1%. At pH 5.5, the cumulative release rate of Ce6 increased slightly to about 13.5%, but it was still relatively low. Surprisingly, under ultrasound irradiation, the cumulative release rate of Ce6 dramatically increased to approximately 57.11%. These results confirmed the positive effect of ultrasound irradiation on the release of Ce6 at the tumor site. In conclusion, these results showed that the Ce6 ultrasound-response release dynamics of CMB NPs was favored sonodynamic therapy (SDT) for tumors.

### *In vitro* ROS generation and TME-triggered SDT enhancement

**Figure 2.**
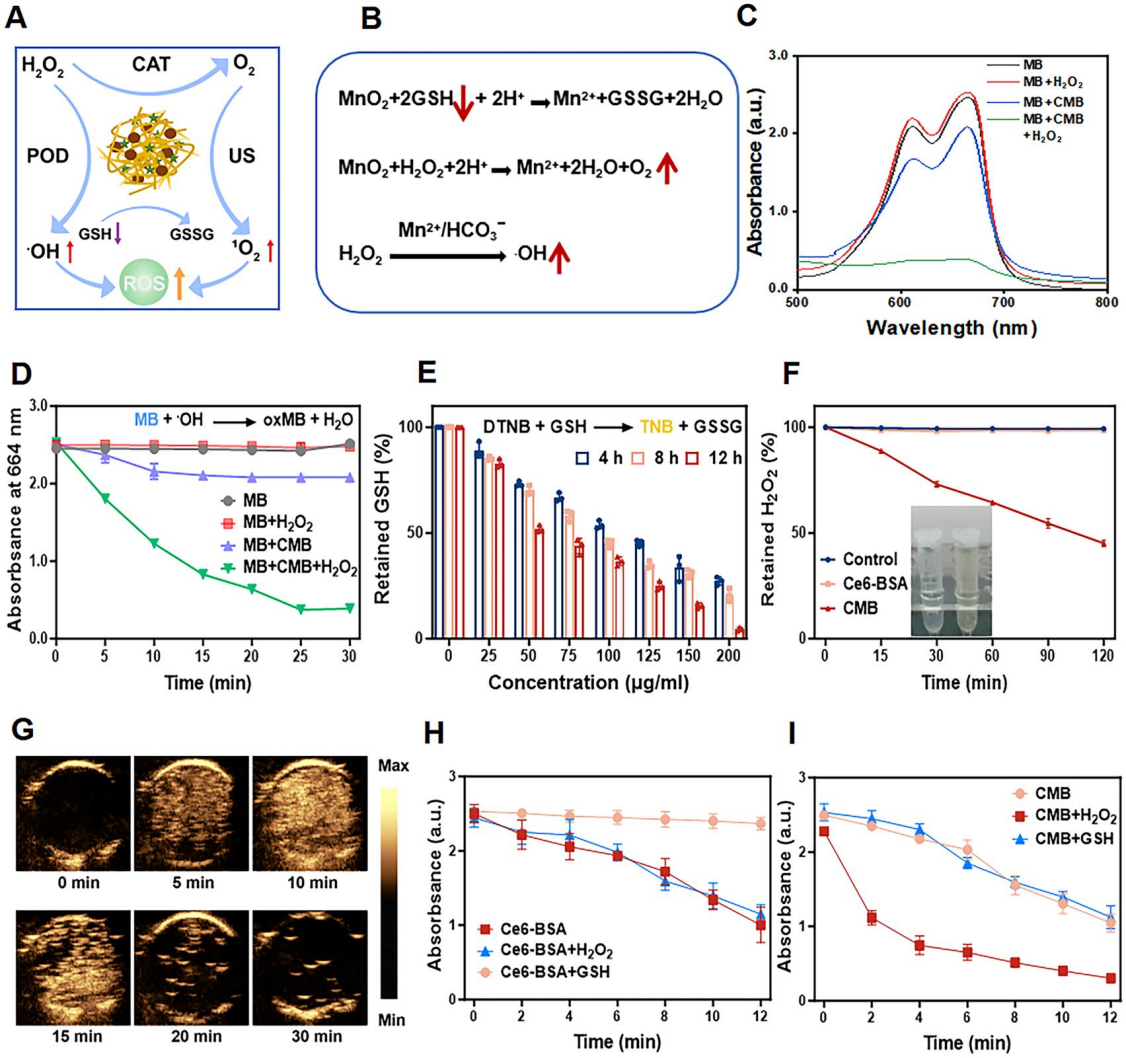
*In vitro* ROS generation and TME-triggered SDT enhancement. (**A**) and (**B**) Schematic illustration of ROS generation and TME-triggered SDT enhancement. (**C**) The Fenton catalytic reaction activity mediated by CMB NPs. (**D**) The degradation of MB mediated by CMB NPs over time. (**E**) The consumption of GSH by CMB NPs. (**F**) The CAT-like enzyme activity of CMB NPs. (**G**) The characteristics of O_2_ production by ultrasound imaging technology. (**H**) TME-triggered the production of ^1^O_2_ mediated by Ce6-BSA NPs. (**I**) TME-triggered the production of ^1^O_2_ mediated by CMB NPs.

As shown in Figure 2A and B, CMB NPs increased TME-triggered ROS production. To verify the ability of CMB NPs to catalyze the generation of ⋅OH, we conducted an *in vitro* experiment using methylene blue (MB) degradation assay to assess the ROS generation capacity. MB has a distinct absorption peak at 652 nm, and the ⋅OH generated in the reaction can convert the blue MB into the colorless oxMB. Therefore, the generation of ⋅OH can be reflected by measuring the residual amount of MB. Therefore, as shown in the Figure 2C, the UV–Vis absorption spectra of MB in NaHCO_3_ buffer after different treatments were measured. The change in MB absorbance in the blank and H_2_O_2_ alone groups could be neglected. In contrast, the MB absorbance slightly decreased in the CMB NPs group, while the decrease was most significant in the CMB + H_2_O_2_ group, where the absorbance was almost depleted. In addition, the degradation of MB over time is shown in the Figure 2D. As the reaction time increased, the MB absorbance in the blank and H_2_O_2_ alone groups did not show significant changes. In the CMB NPs group, the MB absorbance slightly decreased in the first 10 min, and then, stabilized after 10 min. As expected, the characteristic absorption peak of MB at 652 nm in the CMB + H_2_O_2_ group continuously decreased over 30 min, indicating that MB was undergoing continuous degradation. These results suggested that CMB NPs could continuously generate lethal ROS at the tumor site.

GSH is a naturally occurring antioxidant that can react with intracellular oxidants to prevent damage to cell mitochondria and membrane, thereby shielding tumor cells from ROS-induced cell death. Therefore, the consumption of oxidants generated by SDT due to the overexpression of GSH in the TME is a key factor to consider in improving the efficiency of SDT-induced ferroptosis. Then, we used the colorless probe 5,5'-dithiobis(2-nitrobenzoic acid) (DTNB) to assess the consumption of GSH. The sulfhydryl group in the probe reacted with the sulfhydryl group in GSH to form a yellow product, 5-thio-2-nitrobenzoic acid (TNB), which showed a prominent absorption peak at 412 nm (Figure 2E). We were surprised to find that the consumption of GSH by CMB NPs increased over time at the same concentration. Moreover, the amount of GSH consumed increased gradually with increasing concentrations of CMB NPs. These findings confirmed the excellent ability of CMB NPs to consume GSH, indicating their great potential in enhancing the efficacy of SDT.

Catalase-like (CAT-like) enzyme activity refers to the catalytic ability of enzymes similar to catalase (CAT), with the primary characteristic of catalyzing the decomposition of hydrogen peroxide (H_2_O_2_) into water (H_2_O) and O_2_ [46]. In an acidic environment, H_2_O_2_ reacts with Ti(SO_4_)_2_ to form a yellow precipitate, which has a characteristic absorption peak at 412 nm. Therefore, the Ti(SO_4_)_2_-based colorimetric analysis is used to determine the H_2_O_2_ content in the solution, further evaluating the CAT-like activity of CMB NPs. As shown in Figure 2F, the results indicated that the characteristic peak of CMB NPs decreased sharply with the increase in time, showing that H_2_O_2_ in solution was degraded by CMB NPs and that MnO_2_ could effectively catalyze H_2_O_2_ decomposition. On the other hand, the efficiency of O_2_ generation by CMB NPs *in vitro* is also an important indicator for verifying the CAT-like activity of CMB NPs. As shown in the recorded image, the left side represented the Ce6-BSA group and the right side represented the CMB NPs group. The results suggested that CMB NPs could catalyze the production of abundant O_2_ from H_2_O_2_. We used the O_2_ fluorescence probe Ru(dpp)_3_Cl_2_ and ultrasound imaging technology for validation. Since Ru(dpp)_3_Cl_2_ exhibits fluorescence quenching in the presence of O_2_ [47], changes in its fluorescence intensity can indirectly reflect the efficiency of O_2_ generation. As shown in Supplementary Figure S5, the results indicated that as the reaction time increased, the fluorescence intensity of Ru(dpp)_3_Cl_2_ significantly decreased, particularly within the first 15 min. This suggested that the O_2_ generation level increased rapidly with the reaction time. In addition, O_2_, as an ultrasound contrast agent, can enhance ultrasound imaging. Therefore, ultrasound imaging technology can be used to visualize the O_2_ generation [48]. As shown in the Figure 2G and Supplementary Figure S6, no ultrasound signal was observed in the H_2_O_2_ solution without the addition of CMB NPs suspension. In contrast, after the addition of CMB NPs suspension, rapidly enhanced ultrasound signals were detected in the H_2_O_2_ solution, indicating that ultrasound imaging could be used to monitor O_2_ generation. Moreover, as the reaction time increased, the changes in ultrasound signals were similar to those observed with the Ru(dpp)_3_Cl_2_ fluorescence probe. These results suggested that CMB NPs exhibited excellent O_2_ generation performance in the H_2_O_2_-rich TME.

1,3-Diphenylisobenzofuran (DPBF) is a singlet oxygen probe with a characteristic absorption peak at 415 nm, and is commonly used to study the generation of ^1^O_2_ [49]. The CMB NPs suspension was mixed with the DPBF probe, and the change in absorbance at 415 nm was monitored as the ultrasound exposure time was extended, to investigate the ^1^O_2_ generation performance. As shown in Supplementary Figure S7, the presence of CMB NPs significantly decreased DPBF absorption with increasing ultrasound irradiation time, which indicated excellent ^1^O_2_ generation capacity. Moreover, H_2_O_2_ and GSH were added to Ce6-BSA or CMB NPs to investigate the generation of ^1^O_2_. As shown in Figure 2H and I, regardless of the presence or absence of H_2_O_2_, no significant difference was observed in the absorbance change at 415 nm for Ce6-BSA NPs under ultrasound irradiation. However, in the presence of H_2_O_2_, the absorbance at 415 nm for CMB NPs under ultrasound irradiation significantly decreased. The results indicated that the presence of H_2_O_2_ enhanced the ^1^O_2_ production efficiency of CMB NPs, possibly due to the reaction between CMB NPs and H_2_O_2_ generating O_2_, thereby increasing the yield of ^1^O_2_. In contrast, Ce6-BSA NPs did not react with H_2_O_2_ to generate O_2_, and therefore, did not produce more ^1^O_2_. In addition, GSH was added to Ce6-BSA or CMB NPs to investigate the generation of ^1^O_2_. The presence of GSH decreased the ^1^O_2_ production efficiency of Ce6-BSA NPs under ultrasound irradiation compared with that of Ce6-BSA NPs without GSH, implying that ^1^O_2_ may be consumed by GSH. In contrast, GSH could be consumed by CMB NPs, such that whether in the presence or absence of GSH, a large amount of ^1^O_2_ would be produced by CMB NPs under ultrasound irradiation. Therefore, being able to respond to the overexpression of H_2_O_2_ and GSH in the TME, CMB NPs enhanced the SDT effect and created great potential for SDT-induced ferroptosis.

### *In vitro* cellular uptake and cytotoxicity research on CMB NPs

**Figure 3.**
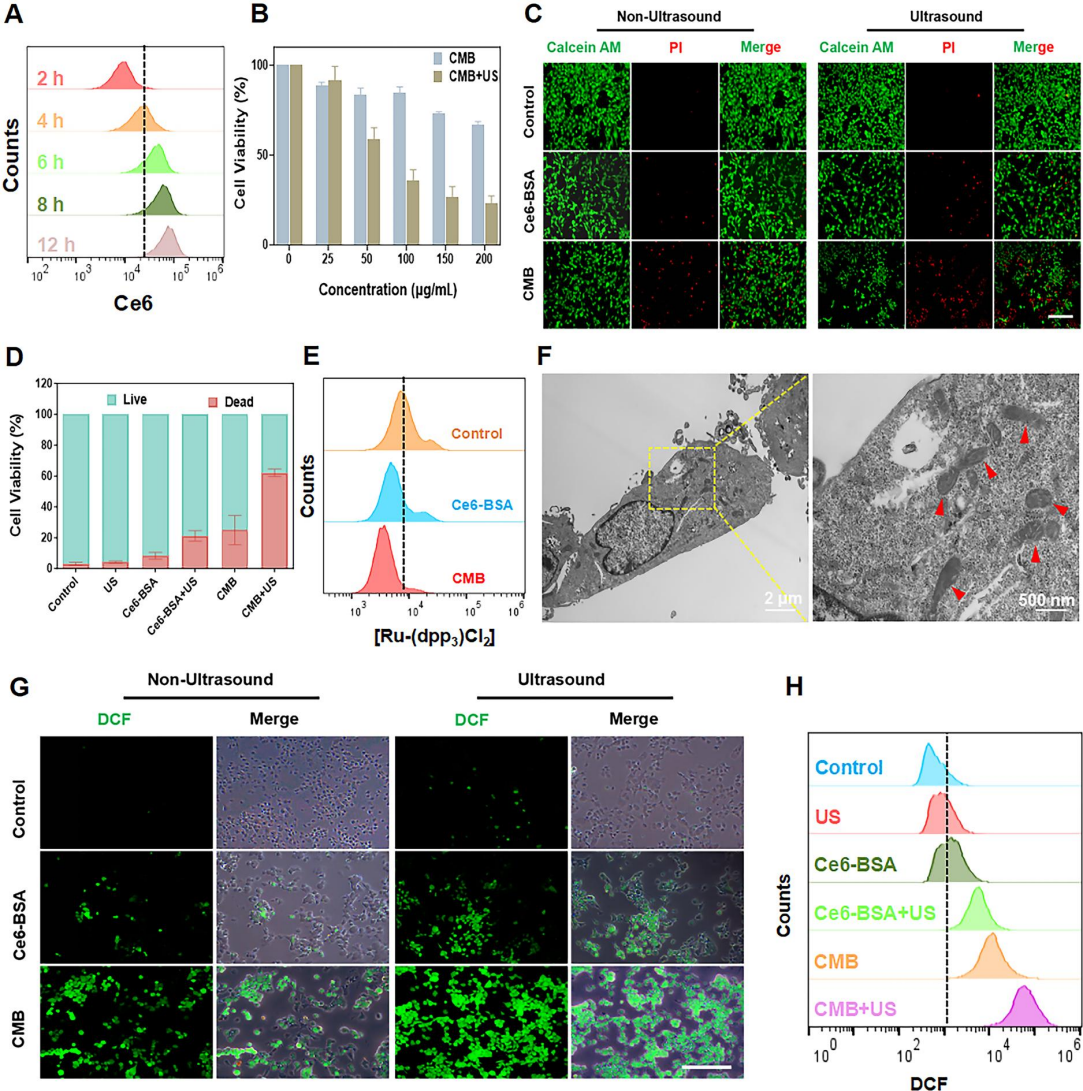
The detection of ferroptosis induced by CMB NPs upon ultrasound irradiation. (**A**) Cellular uptake of CMB NPs by flow cytometric. (**B**) The cytotoxicity of CMB NPs with or without ultrasound irradiation at different concentrations. (**C**) Calcein-AM/PI analysis after different treatments. Scale bar: 100 μm. (**D**) Statistical results of Calcein-AM/PI analysis. (**E**) The validation of intracellular O_2_ by flow cytometric. (**F**) Representative bio-TEM images of the mitochondrial membrane density (red arrow) in 4T1 cells. Left: Incubation with CMB NPs and exposure to ultrasound irradiation. Right: Partially enlarged view of left. (**G**) Production of ROS in 4T1 cells of different groups by fluorescence microscopy. Scale bar: 100 μm. (**H**) Production of ROS in 4T1 cells of different groups by flow cytometric.

We next investigated the intracellular behavior of CMB NPs endocytosis by 4T1 cells. Using the fluorescent properties of Ce6, we analyzed the 4T1 cells incubated with CMB NPs by flow cytometry and found that the CMB NPs signal inside the cells gradually increased with the extension of the incubation time (Figure 3A). The results showed that CMB NPs could be internalized by 4T1 cells, which could be attributed to their good biocompatibility. Subsequently, the safety of CMB NPs under non-ultrasound conditions was tested. The CCK-8 assay results showed that after treatment with CMB NPs at concentrations reaching as much as 200 μg/mL, the viability of HUVECs was greater than 80%, which confirmed the good biocompatibility of CMB NPs (Supplementary Figure S8). Next, the cytotoxicity of CMB NPs toward 4T1 cells with or without ultrasound irradiation at different concentrations was evaluated. As shown in Figure 3B, CMB NPs exhibited dose-dependent cytotoxicity without ultrasound irradiation; in contrast, the cytotoxicity of CMB NPs at an equivalent dosage significantly increased under ultrasound irradiation. Further assessment of cell viability was conducted using Calcein-AM/PI staining. As shown in Figure 3C, the proportion of PI-stained cells in the CMB+US group was obviously higher than in the other groups. Fluorescence quantification analysis also indicated that the percentage of dead cells in the CMB+US group was significantly higher than in the other groups (Figure 3D). These results suggested that CMB NPs significantly enhanced the ability to induce tumor cell death under ultrasound irradiation, thereby achieving the optimal antitumor therapeutic effect.

### Validation of intracellular O_2_

The continuous rapid proliferation of tumor cells results in the incomplete formation of blood vessel walls, which causes an inadequate supply of O_2_, leading to a hypoxic microenvironment in the tumor tissue. However, highly dependent on O_2_ concentration is the SDT effect, and often, the hypoxic state of the tumor leads to low SDT efficiency. Furthermore, an oxygen-consuming process is the SDT treatment, and as a result, the consumption of O_2_ further damages the tumor microvascular structure, exacerbating the tumor’s hypoxic condition [26]. Therefore, generating O_2_  *in situ* at the tumor site to alleviate the hypoxic condition is a crucial strategy for improving the efficacy of SDT. To further demonstrate the ability of CMB NPs to generate O_2_ within cells, we used the fluorescent probe Ru(dpp)_3_Cl_2_ to detect the O_2_ levels inside the cells. As shown in Figure 3E, compared to the control group, after co-incubation of 4T1 cells with CMB NPs, the red fluorescence inside the cells significantly decreased, indicating that CMB NPs generated a sufficient amount of O_2_ within the cells, thereby alleviating the hypoxic condition of the tumor cells.

### Cascade-enhanced strategy for boosting ferroptosis

The occurrence of ferroptosis is usually accompanied by significant changes in mitochondria, with mitochondrial damage being one of the key markers in the process of ferroptosis [15]. Specifically, characterized by mitochondrial membrane bubbling and rupture, increased mitochondrial membrane density, reduced or absent mitochondrial cristae and mitochondrial shrinkage, are the morphological changes. According to the magnified bio-TEM images, the CMB + US group exhibited increased mitochondrial membrane density (Figure 3F) and a loss in the number of mitochondrial cristae (Supplementary Figure S9), which is similar to the characteristic changes in mitochondrial damage associated with ferroptosis. The mechanism proposed in this study for ultrasound-irradiated CMB NPs enhancing ferroptosis is as follows: (1) the presence of high levels of ROS causes lipid peroxidation, thereby inducing ferroptosis; (2) GSH consumption by CMB NPs inactivates GPX4 and induces lipid peroxidation, followed by ferroptosis; and (3) a high level of ROS can cause autophagy, thus, enhancing ferroptosis.

**Figure 4.**
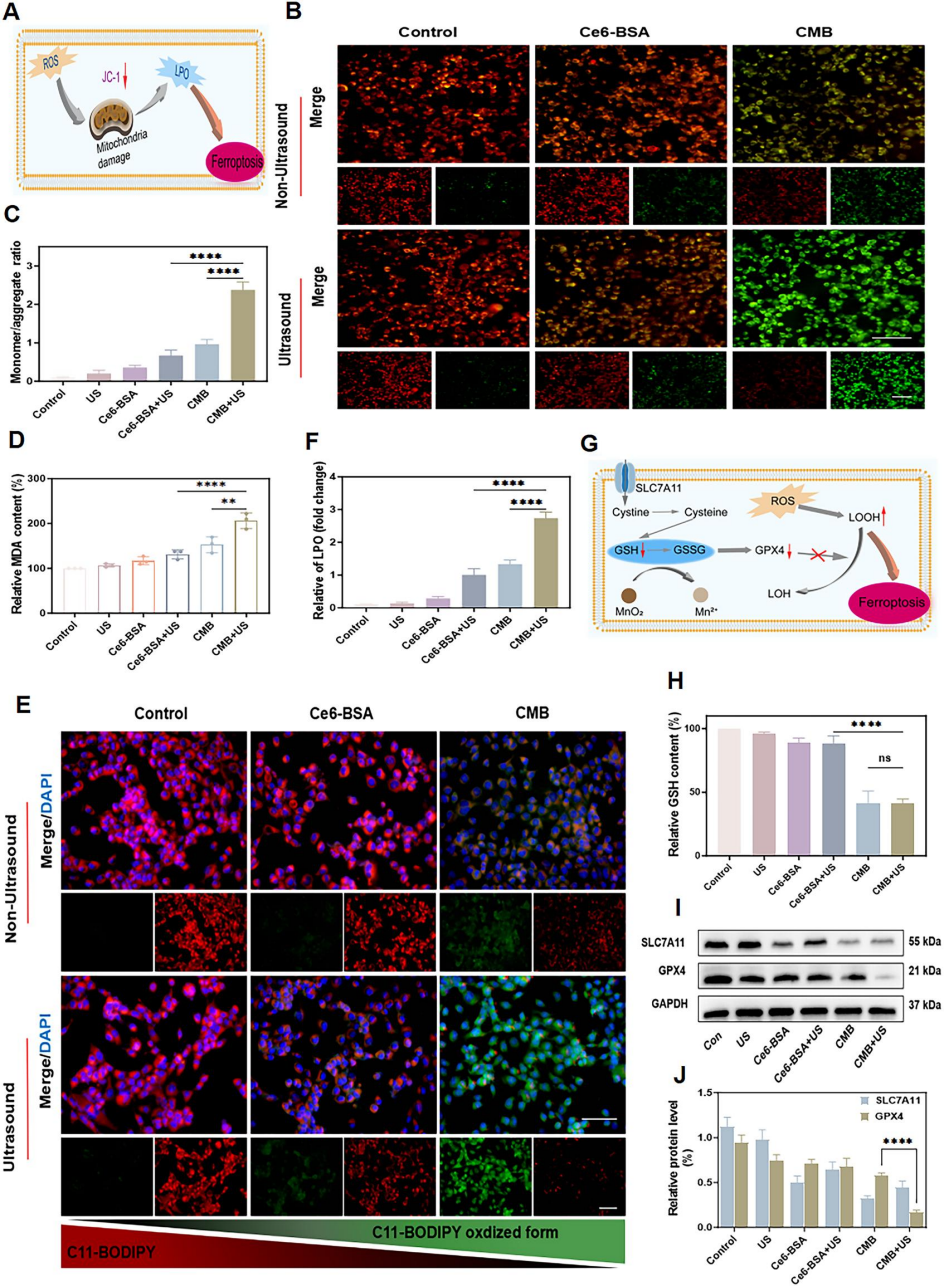
The generation of mitochondrial ROS leading to LPO and SLC7A11–GSH–GPX4 axis system regulation by CMB NPs. (**A**) Schematic illustration of cellular ferroptosis induced by LPO accumulation. (**B**) Fluorescence microscopic imaging of 4T1 cells stained with JC-1 under different treatments. Scale bar: 100 μm. (**C**) The monomer/aggregate fluorescence intensity ratio of JC-1. (**D**) Relative MDA content under different treatments. (**E**) Lipid peroxides (scale bar: 100 μm) and (**F**) relative of LPO (fold change) of 4T1 cells stained with C11-BODIPY after varied treatments. (**G**) Schematic illustration of SLC7A11–GSH–GPX4 axis system defending against ferroptosis. (**H**) The GSH-consumption performance of CMB NPs. (**I**) Western blot analysis of the expression level of SLC7A11 and GPX4. (**J**) SLC7A11 and GPX4 protein expressions in 4T1 cells following varying interventions. The statistical analysis of the data was performed using one-way ANOVAs. Data are presented as mean ± SD, ns: no significant difference, **P < 0.01, ****P < 0.0001.

1. The increased generation of ROS will cause damage to the cell membrane, exacerbate mitochondrial damage and lead to LPO and the occurrence of ferroptosis. The ability of CMB NPs to generate ROS under ultrasound irradiation was further investigated using 2',7'-dichlorodihydrofluorescein diacetate (DCFH-DA). Once DCFH-DA reacts with ROS, it is oxidized and exhibits green fluorescence. As shown in Figure 3G, under the condition of ultrasound irradiation alone, there was no significant increase in DCF fluorescence, indicating that ultrasound exposure did not cause significant damage to the cells. Moreover, after incubation with Ce6-BSA NPs, slice- and star-shaped DCF fluorescence was observed with and without ultrasound irradiation, respectively, suggesting that Ce6-BSA NPs produced a low level of ROS under ultrasound irradiation. Interestingly, an increase in flake fluorescence was detected after incubation with CMB NPs; however, large-scale DCF fluorescence was observed under ultrasound irradiation, indicating that CMB NPs produced considerable amounts of ROS under ultrasound irradiation. In addition, the prominent ability of CMB NPs to induce ROS in 4T1 cells under ultrasound irradiation was confirmed by flow cytometry (Figure 3H). The high level of ROS production could have been due to the catalysis of H_2_O_2_ by CMB NPs, which generated ·OH, and the production of O_2_ alleviated hypoxia, thereby improving the efficiency of SDT in generating ^1^O_2_. Additionally, the downregulation of GSH by CMB NPs may have prevented excessive GSH from consuming ^1^O_2_, further promoting the accumulation of ROS. A high levels of ROS can induce oxidative damage to mitochondria, leading to the disruption of mitochondrial membrane potential, which plays an important role in triggering ferroptosis (Figure 4A).

Furthermore, JC-1, as a fluorescent dye for assessing mitochondrial membrane potential, aggregates and gives rise to the emission of red fluorescence, indicative of a normal mitochondrial membrane potential. However, in instances where the mitochondrial membrane potential undergoes depolarization, JC-1 persists in its monomeric form, emitting green fluorescence, which is frequently utilized as a diagnostic marker of mitochondrial dysfunction. The JC-1 staining results are shown in Figure 4B. The control group, ultrasound group and Ce6-BSA group mainly exhibited red fluorescence, indicating normal mitochondrial status. The Ce6-BSA + US group and CMB group showed an increase in green fluorescence, while the red fluorescence signal correspondingly weakened, suggesting mitochondrial damage. However, the CMB + US group predominantly displayed green fluorescence with the strongest intensity, indicating the greatest mitochondrial damage. The fluorescence quantitative analysis, as shown in the Figure 4C, indicated that the CMB + US group had the highest monomer/aggregate ratio, confirming that mitochondrial damage was most severe. Excessive ROS not only cause mitochondrial damage but also exacerbate mitochondrial injury through LPO, thereby promoting ferroptosis in cancer cells.

With strong oxidative properties, ROS can attack the unsaturated fatty acids in cell membranes, triggering LPO. Malondialdehyde (MDA), a crucial byproduct of LPO, serves as a well-established biomarker for evaluating the degree of oxidative damage within cells or tissues. The results demonstrated that the MDA levels in the CMB + US group were markedly elevated compared to those observed in all other experimental groups, indicating a pronounced increase in oxidative stress (Figure 4D), indicating that the constructed boosting system had an excellent ability to induce ferroptosis. Furthermore, the intracellular LPO levels can be assessed using the dual-fluorescence probe C11-BODIPY581/591. When LPO accumulates within the cell, the BODIPY structure in C11-BODIPY581/591 becomes oxidized, causing its fluorescence to shift from red (580 nm) to green (510 nm). As shown in Figure 4E, compared with that of the other groups, the CMB + US group exhibited the strongest green fluorescent signal. The fluorescence quantitative analysis results also showed the same trend (Figure 4F). This confirmed that CMB NPs, under ultrasound induction, enhanced LPO accumulation and demonstrated the potential to induce ferroptosis.

**Figure 5.**
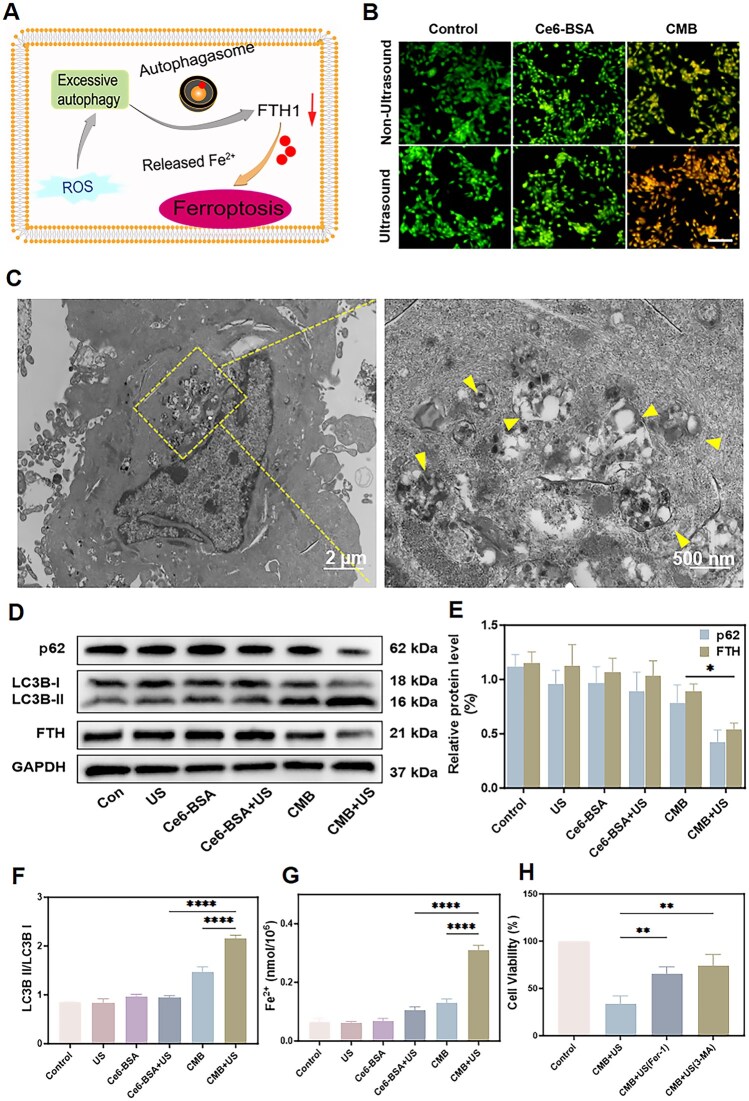
Autophagy regulation by CMB NPs. (**A**) Schematic illustration of cellular autophagy-enhanced ferroptosis induced by CMB NPs. (**B**) Fluorescence microscopic imaging of 4T1 cells stained by AO after different treatments. Scale bar: 100 μm. (**C**) Representative bio-TEM images of the autophagosomes (yellow arrow) in 4T1 cells. Left: Incubation with CMB NPs and exposure to ultrasound irradiation. Right: Partially enlarged view of left. (**D**) Western blot analysis of the expression level of p62, LC3B and FTH. (**E**) p62 and FTH protein expressions in 4T1 cells following varying interventions. (**F**) LC3B II/LC3B I protein expressions in 4T1 cells. (**G**) The Fe^2+^ levels in 4T1 cells following varying interventions. (**H**) Percentual cell viability of 4T1 cells upon preincubation with cell death inhibitors Fer-1 (50 μM), and 3-MA (100 μM) followed by incubation with CMB NPs under ultrasound irradiation. The statistical analysis of the data was performed using one-way ANOVAs. Data are presented as mean ± SD, ns: no significant difference, *P < 0.05, **P < 0.01, ****P < 0.0001.

(2) The SLC7A11-GSH-GPX4 axis is a key molecular pathway involved in the regulation of ferroptosis. When this axis is disrupted (such as by SLC7A11 inhibition, GSH depletion or GPX4 inactivation), cells become more susceptible to ferroptosis due to the accumulation of oxidative damage (Figure 4G). Since preliminary experiments had validated that CMB NPs exhibited superior GSH consumption capability, a GSH detection kit was used to assess the GSH levels in 4T1 cells. There was no significant difference in intracellular GSH consumption after treatment with CMB (approximately 58.9%) or CMB + US (approximately 58.5%) (Figure 4H). These results confirmed that the excellent GSH consumption performance of CMB is not affected by ultrasound irradiation. Glutathione peroxidase 4 (GPX4) is a key enzyme that uses GSH to transform toxic LOOH into nontoxic phospholipids, preventing the accumulation of LPO and inhibiting ferroptosis. Additionally, responsible for transporting cystine, a key precursor of glutathione (GSH), into the cell is System Xc- (SLC7A11), a cysteine/glutamate reverse transport protein. The Western blot (WB) analysis was used to assess the expression levels of GPX4 and SLC7A11 inside the cells. The results showed that (Figure 4I and J), compared to the other groups, the expression levels of GPX4 and SLC7A11 in the CMB + US group were significantly reduced, suggesting that the combination of CMB NPs and ultrasound irradiation effectively inhibited the SLC7A11-GSH-GPX4 axis’s defense against ferroptosis, providing a promising application for boosting ferroptosis in tumor cells.

(3) Autophagy-mediated ferritin degradation disrupts the cell’s iron storage and homeostasis. When ferritin is degraded by autophagy, the released free iron ions significantly increase the intracellular iron concentration, triggering ferroptosis (Figure 5A). To assess the effect of CMB NPs under ultrasound irradiation on 4T1 cell autophagy, we utilized acridine orange (AO), a fluorescent dye that is detects autophagy levels. AO is sensitive to pH and emits green fluorescence in the cytoplasm but red fluorescence in acidic vesicular organelles such as autophagosomes. Figure 5B showed that, compared with that in the other groups, an increase in bright orange color was detected in the CMB + US group. This observation confirmed that CMB NPs induced autophagy in cancer cells under ultrasound irradiation. In addition, bio-TEM is widely recognized as a benchmark method for observing the ultrastructural features of autophagosomes. Figure 5C and Supplementary Figure S10 showed the atypical accumulation of autophagosomes and other autophagic vesicles in tumor cells treated with the ferroptosis boosting system, suggesting a potent ability to induce autophagy.

In light of these findings, we further confirmed the potential autophagy pathways influenced by CMB NPs under ultrasound irradiation through WB analysis. When the autophagic process is active, low is the intracellular level of p62, as p62 is an important marker of autophagic activity and its expression level is negatively correlated with autophagy. The results, as shown in the Figure 5D and E, indicated that the expression level of p62 was significantly downregulated in the CMB + US group. The results also verified that the CMB NPs induced autophagy in cancer cells under ultrasound irradiation. Moreover, typically increased is the level of microtubule-associated protein 1 light chain 3 isoform II (LC3-II) when autophagy is active, as microtubule-associated protein 1 light chain 3 beta (MAP1LC3B/LC3), an important component of the autophagosome membrane, is converted from its precursor form (Microtubule-associated protein 1 light chain 3 isoform I, LC3-I) to LC3-II during the autophagic process. As shown in Figure 5D and F, the CMB + US group significantly promoted the turnover of LC3-I to LC3-II, in contrast to the other groups. Recent studies have shown that the degradation of ferritin through autophagic processes can lead to a decrease in intracellular FTH1 levels, thereby increasing the levels of free iron in the cells and promoting ferroptosis in tumor cells [50]. FTH1 expression decreased significantly in the CMB + US group, as it can induce potent autophagy, whereas the other groups minimally decreased FTH1 (Figure 5D and E). Furthermore, autophagic degradation of ferritin increases the levels of free iron ions (especially Fe^2+^) in the cells. Therefore, it is essential to detect the intracellular Fe^2+^ levels. As expected, the Fe^2+^ levels were significantly elevated in the cancer cells of the CMB + US group (Figure 5G). In conclusion, the above results provide reliable evidence for the activation of autophagy by CMB NPs under ultrasound irradiation to enhance the ferroptosis pathway, deepening the understanding of the potential mechanism of the constructed ferroptosis boosting system in anti-tumor therapy.

To further confirm the cytotoxicity of ferroptosis enhanced by autophagy induced by CMB under ultrasound irradiation, the autophagy-specific inhibitor 3-methyladenine (3-MA) and the ferroptosis inhibitor ferrostatin-1 (Fer-1) were utilized as targeted modulators of autophagy and ferroptosis, respectively. Subsequently, the cytotoxic effects on the cells were quantitatively evaluated through the CCK-8 assay, which measures cell viability. The results showed that after 3-MA treatment, the cell cytotoxicity induced by CMB NPs was effectively reduced by approximately 40%, and after Fer-1 treatment, the cytotoxicity was effectively reduced by about 31% (Figure 5H), suggesting that CMB NPs under ultrasound irradiation exhibit excellent autophagy-enhanced ferroptosis properties.

### *In vivo* anticancer performance and mechanism analysis

**Figure 6.**
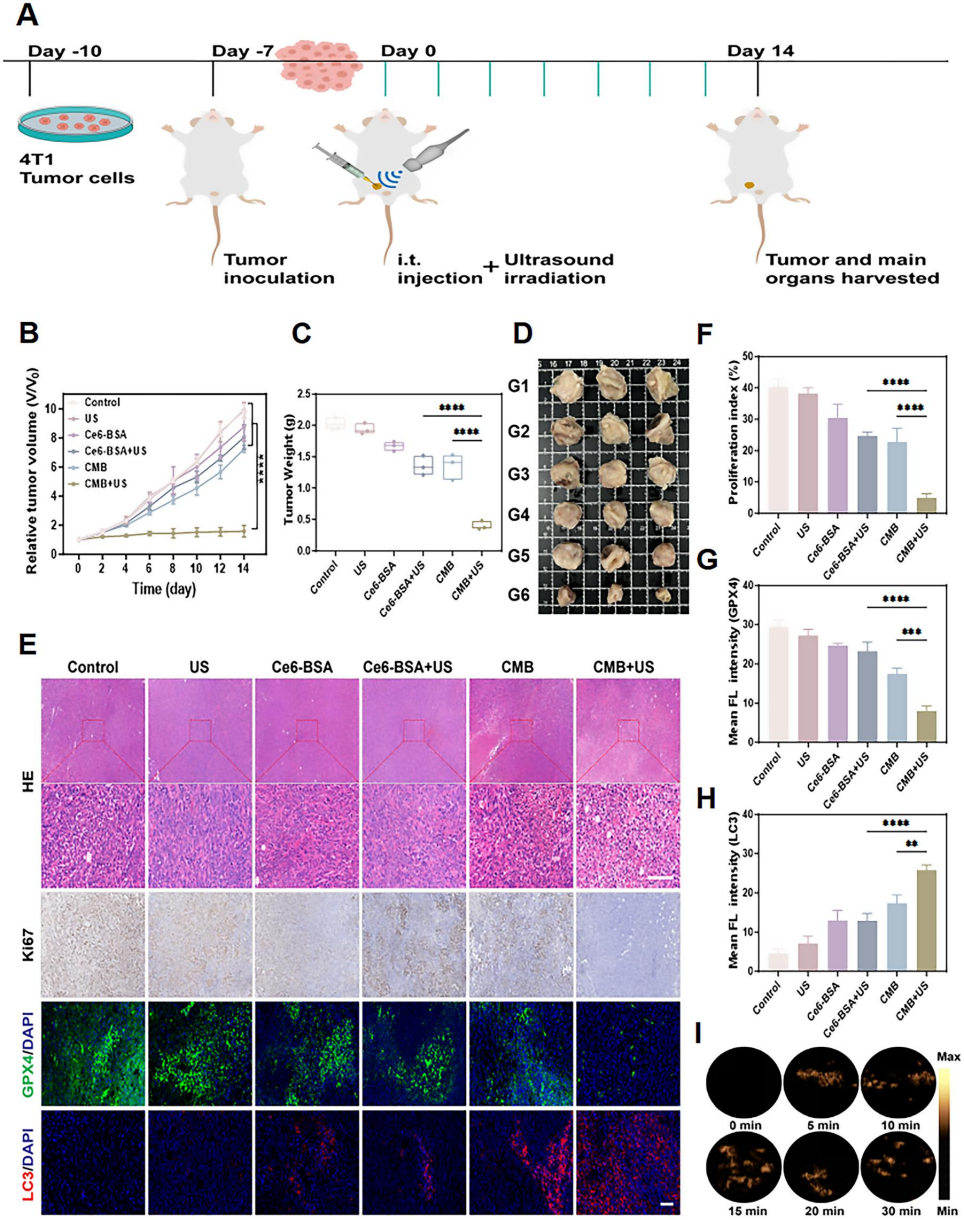
*In vivo* therapeutic efficacy of CMB NPs. (**A**) *In vivo* experimental treatment plan. (**B**) Photos of tumors. (G1: Control, G2: US, G3: Ce6-BSA, G4: Ce6-BSA+US, G5: CMB, G6: CMB + US). (**C**) Tumor growth curve of each group. (**D**)Tumor weights after different treatments. (**E**) Immunohistochemical (HE and Ki67) and immunofluorescence (GPX4 and LC3) analyses of tumor tissue sections from different treated mice. Scale bar: 100 μm. (**F**) Proliferation index of tumor tissue. (**G**) Mean fluorescence intensity of GPX4. (**H**) Mean fluorescence intensity of LC3. (**I**) The generation of O_2_  *in vivo* of CMB NPs. The statistical analysis of the data was performed using one-way ANOVAs. Data are presented as mean ± SD, **P < 0.01, ***P < 0.001, ****P < 0.0001.

Based on the excellent therapeutic effect of CMB NPs under ultrasound irradiation *in vitro*, the anti-tumor effect of the constructed ferroptosis boosting system *in vivo* was evaluated. The method for establishing the Balb/c mice bearing 4T1 tumor model and the detailed treatment protocol are shown in Figure 6A. Since intratumoral injection can achieve greater drug accumulation at the tumor site compared to tail vein injection, to better understand the *in vivo* antitumor mechanism of the constructed ferroptosis boosting system, we chose intratumoral injection. Ultrasound irradiation was selected to be performed immediately after intratumoral injection, targeting the tumor site. The tumor volume in the CMB + US group mice was the smallest, indicating that it has a strong antitumor effect (Figure 6B). The tumor volume in groups Control, US and Ce6-BSA increased rapidly, while in groups Ce6-BSA + US and CMB, the tumor volume increased more slowly, but still showed a significant increase. Similarly, the tumor weight in the CMB + US group was the lowest (Figure 6C). The inhibition rates of the CMB+US group were calculated to be 83.6% and 80.6% based on the mean tumor volume and weight, respectively (Supplementary Figure S11). The same conclusion can be drawn from the tumor morphology images obtained after treatment (Figure 6D). The results demonstrated that CMB NPs, under ultrasound irradiation, exhibited excellent tumor suppression effects. This effect of CMB+US was further assessed via hematoxylin and eosin (HE) staining, immunohistochemical staining and immunofluorescence staining (Figure 6E). The HE results showed that tumor cells in the CMB + US group were the most loosely arranged, suggesting that tumor cell necrosis was significantly increased in this group. As expected, Ki67 staining, a marker of cell proliferation, was lowest in the CMB + US group (Figure 6F). The quantitative Ki67-positive cell assessment revealed that the cell proliferation rate of the CMB+US-treated group was only 4.92%, which was lower than that of the other groups.

As previously described, the ability of the ferroptosis boosting system we constructed to reduce GSH levels and inhibit GPX4 expression has been confirmed both extracellularly and intracellularly. *In vivo*, we used tumor tissue sections to verify the ability of the ferroptosis boosting system to inhibit GPX4. Analysis of animal tumor tissues by GPX4 immunofluorescence staining showed that in the CMB+US group, only star-shaped green fluorescence was observed (Figure 6E and G), indicating that it significantly inhibited the expression of GPX4 and induced ferroptosis in tumor cells. Moreover, immunofluorescence staining analysis of the autophagy-related protein LC3 showed that the CMB+US group exhibited the strongest red fluorescence compared to other groups (Figure 6E and H), indicating the highest expression of LC3 and the most robust autophagic response. Additionally, we utilized ultrasound imaging technology to visualize and assess oxygen generation *in vivo*. After intratumoral injection of CMB NPs in tumor-bearing mice, the ultrasound signals in the tumor tissue rapidly increased over time (Figure 6I and Supplementary Figure S12), indicating effective oxygen production at the tumor site, thereby helping to alleviate the hypoxic conditions in the TME. These results confirmed that the designed boosting system, under ultrasound irradiation, can significantly promote ferroptosis in tumors and possesses a stronger antitumor therapeutic effect.

### Biosafety evaluations

**Figure 7.**
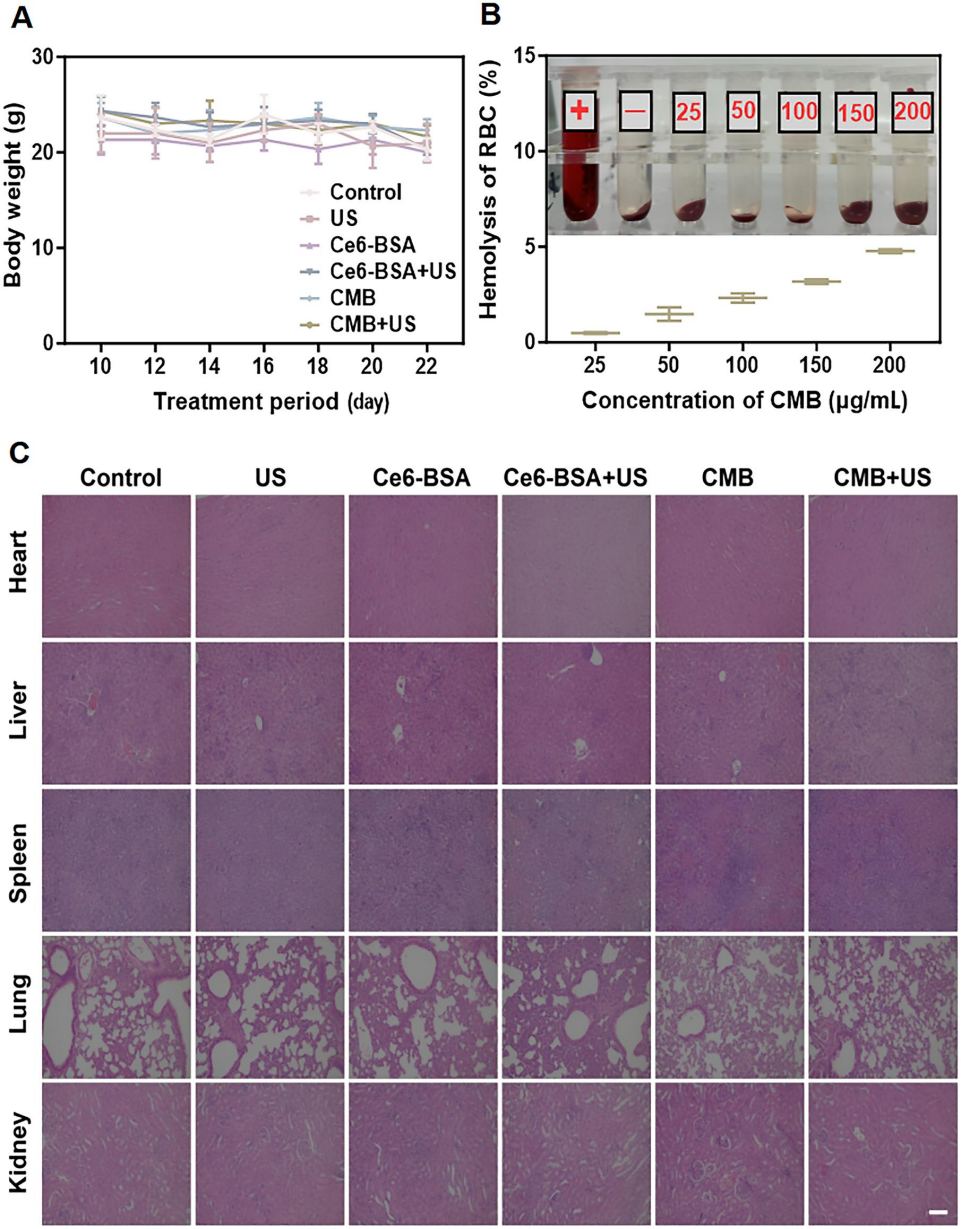
Biosafety evaluations. (**A**) Body weight of mice during treatment. (**B**) Hemolytic capacity of CMB NPs. (**C**) H&E staining of major organs from different groups. Scale bar: 100 μm.

Although intratumoral injection directly targets the tumor site, the drug may still enter the bloodstream through local absorption, potentially affecting other organs or systems. Therefore, evaluating biological safety is crucial, as it helps enhance the likelihood of clinical translation. During the treatment, the body weight changes of tumor-bearing mice were monitored. The results indicated that there were no statistically significant alterations in the body weight of the tumor-bearing mice across all experimental groups (Figure 7A). In addition, the hemolysis assay showed that there was no obvious hemolysis under different concentrations of CMB NPs (Figure 7B). Importantly, histological results of the main organs (heart, liver, spleen, lung and kidney) in the control and treatment groups showed that acute pathological toxicity and adverse reactions during treatment were negligible (Figure 7C). Furthermore, no statistically significant differences were observed in the organ indices, including those of the heart, liver, spleen, lungs and kidneys, when comparing the treatment group to the control group, suggesting that the treatment did not cause notable side effects in the mice (Supplementary Figure S13). These results indicated that CMB NPs have good biological safety, providing convincing impetus for the clinical application of the developed ferroptosis boosting system.

### Blood drug concentration experiment

Since it had been previously verified that Ce6 was successfully loaded into CMB NPs, the concentration of Ce6 in the mouse blood at different time points after injection was subsequently quantitatively assessed to further infer the pharmacokinetic changes of CMB NPs. The results, shown in the Supplementary Figure S14, indicated that after the injection of CMB NPs, the concentration of Ce6 in the mouse blood rapidly increased, peaked 30 min postinjection, and then, quickly decreased. No significant Ce6 residue was detected 6 hr after injection, indicating that CMB NPs were effectively cleared from the mouse body.

### Conclusion

In summary, a cascade-augmented strategy for the treatment of TNBC, which results in excellent ROS generation, superior GSH consumption and potent autophagy, was rationally proposed. The ferroptosis boosting system CMB NPs were developed on a BSA platform with excellent biocompatibility and were loaded with MnO_2_ NPs and Ce6. These engineered ferroptosis boosting system CMB NPs could trigger ⋅OH generation within the tumor sites. After intratumoral injection of CMB NPs, H_2_O_2_/GSH was decreased in the TME and synergistically triggered the production of ^1^O_2_ under ultrasound irradiation. When high levels of ROS are present in tumor sites, the degree of LPO increases to increase cancer cell ferroptosis. Meanwhile, the significant depletion of GSH caused by CMB NPs weakened the function of the SLC7A11-GSH-GPX4 axis that inhibits ferroptosis, resulting in a reduced rate of LPO clearance and increased cancer cell ferroptosis. Furthermore, high levels of ROS effectively induced the autophagic degradation of ferritin in lysosomes of tumor cells, thereby markedly increasing the vulnerability to tumor cells to ferroptosis. Extensive *in vitro* and *in vivo* studies have robustly demonstrated that CMB NPs can significantly generate a ROS storm under ultrasound irradiation, eliminating GSH and inducing an autophagic response to cascade-augmented ferroptosis for the treatment of TNBC. This effective treatment strategy broadens the understanding of nanoplatform development, revealing the potential of ferroptosis boosting system as a potent and potentially transformative therapeutic strategy for the clinical intervention in TNBC.

## Experimental section

### Synthesis of CMB NPs

MnO_2_-BSA NPs were prepared using a simple and rapid method using BSA as a reducing agent and carrier [51]. To be specific, 10 mg of KMnO_4_ and 50 mg of BSA were dissolved thoroughly in deionized water, separately, then, the BSA solution was slowly added to the KMnO_4_ solution in a water bath sonicator. The mixture was subjected to continuous ultrasonication (100 W, 1 h) to yield MnO_2_-BSA NPs. Before being introduced onto the MnO_2_-BSA nanoparticles through covalent bonding, Ce6 was activated to form Ce6-COOH by EDC and NHS in DMSO [52]. Briefly, Ce6 (1 mg), EDC (24 mg) and NHS (48 mg) were dissolved in 10 mL of DMSO and stirred magnetically at room temperature for 4 h to obtain Ce6-COOH. Next, the carboxyl groups of Ce6 were activated using EDC and NHS, allowing Ce6 to form an amide bond with BSA. Purification of Ce6-MnO_2_-BSA nanoparticles (referred to as CMB NPs) was performed by dialysis against deionized water for 24 hours, utilizing a dialysis bag with a 14 kDa molecular weight cutoff (MWCO). Ce6-BSA NPs were prepared by the addition of BSA solution after Ce6 was activated to form Ce6-COOH.

### *In vitro* Ce6 release studies

The Ce6 release study was performed using a dialysis technique *in vitro*. In short, 1 mL of the CMB NPs solution was transferred to a dialysis bag (MWCO = 14 kDa), and the bag was incubated in 15 mL of BSA buffer, stirring at 100 rpm at 37°C. To investigate the effect of different pH values on the release of Ce6, BSA buffer was prepared under different conditions (pH 7.4 or 5.5). Meanwhile, the CMB NPs were sonicated (1 MHz, 50% duty cycle, 2 W/cm^2^) for 10 min, and then, placed in a dialysis bag to investigate the effect of ultrasound irradiation on Ce6 release. At scheduled time points, collected 1 mL of the buffer solution and measured the Ce6 release content using UV−Vis spectrophotometry. At the same time, added 1 mL of the same buffer solution to replenish the buffer.

### MB assay

An MB degradation assay was applied to detect the ability of the CMB NPs to generate **·**OH. Specifically, MB (10 μg/mL), H_2_O_2_ (10 mM) and CMB NPs (10 mg/mL) were mixed by different combination (MB, MB + H_2_O_2_, MB + CMB, MB + CMB + H_2_O_2_). All groups were incubated in a NaHCO_3_ (25 mM) buffer solution for 30 min. The degradation of MB was monitored based on the change in the ultraviolet characteristic absorption peak at 664 nm, further evaluating the **·**OH generation performance.

### GSH consumption assay

The GSH depletion capacity of the CMB NPs was detected using a 5,5'-Dithiobis(2-nitrobenzoic acid) (DTNB) method. CMB NPs (10 mg/mL) were coincubated with GSH (0.5 mM). The concentration-dependent (25, 50, 75, 100, 125, 150 and 200 μg/mL) and time-dependent (4, 8 and 12 h) effects on GSH consumption were investigated. At the specified time points, the mixture was added with DTNB (0.5 mM) reagent, and finally, measured the remaining GSH content using UV−Vis spectrophotometry at a wavelength of 412 nm.

### CAT-like activity assay

The CAT-like activity of CMB NPs was bestowed by Ti(SO_4_)_2_ colorimetry assay. Briefly, the Ti(SO_4_)_2_ working solution was obtained by adding 1.33 mL of 24% Ti(SO_4_)_2_ and 8.33 mL of H_2_SO_4_ to 50 mL of water and mixing thoroughly. Ce6-BSA NPs (10 mg/mL) or CMB NPs (10 mg/mL) and H_2_O_2_ (1 mM) were mixed in PBS buffer. The absorbance at 412 nm of each solution was measured using an UV−Vis spectrophotometer after incubation at room temperature for 120 min.

The fluorescence probe Ru(dpp)_3_Cl_2_ was used as an indicator to measure the evolution of O_2_. One hundred microliters of CMB NPs suspension and 10 μl of ethanol-dissolved Ru(dpp)_3_Cl_2_ solution (1 mM) were transferred to a 96-well microplate reader. After the addition of H_2_O_2_ (1 mM), the fluorescence intensity of Ru(dpp)_3_Cl_2_ was measured at different time points (0, 5, 10, 15, 20, 30 min).

Hundred microliter of H_2_O_2_ (1 mM) was added to 5 mL of deionized water to prepare the H_2_O_2_ solution. Then, CMB NPs suspension was added, and real-time observation and image signal acquisition were performed using ultrasound imaging technology. The echo intensity related to the ultrasound images was assessed with ImageJ software.

### *In vitro*  ^1^O_2_ generation and TME-triggered SDT enhancement

The generation of singlet oxygen after *in vitro* ultrasound irradiation of CMB NPs was measured using 1,3-diphenylisobenzofuran (DPBF) singlet oxygen probe. After mixing CMB NPs (10 μg/mL) with the DPBF solution (20 μmol/L) dissolved in DMSO, ultrasound irradiation (1 MHz, 2 W/cm^2^, 50% duty cycle) was applied every 2 min. The absorption of the mixed solution at 415 nm was then measured using a UV–Vis spectrophotometer. Additionally, Ce6-BSA (10 μg/mL) or CMB NPs (10 μg/mL) with or without H_2_O_2_ or GSH were treated with ultrasound irradiation, and the absorbance of the mixed solution at 415 nm was then measured.

## Supplementary Material

rbaf042_Supplementary_Data

## Data Availability

Data will be made available on request.
