# Supplementary material for: Ferroptosis boosting system based on a sonodynamic therapy cascade-augmented strategy for triple-negative breast cancer therapy
Source: Regen Biomater. 2025 May 20;12:rbaf042. doi: 10.1093/rb/rbaf042 (PMC12188201; doi:10.1093/rb/rbaf042)
Supplement: rbaf042_Supplementary_Data [file rbaf042_supplementary_data.zip › Polishing Certificate.pdf]

Editing Certificate

This document certifies that the manuscript

Ferroptosis boosting system based on a sonodynamic therapy cascade-augmented strategy for triple-negative breast cancer therapy

prepared by the authors

Ju-Ying Zhang,Han-Mei Li,Li-Tao Ye,Yi-Han Leng,Xiao-Qing Wang,You Yang,Qiong Jiang,Lin-Li Feng,Ling Li,Yang Li,Jin-hong Yu

was edited for proper English language, grammar, punctuation, spelling, and overall style by one or more of the highly qualified English speaking editors at AJE.

This certificate was issued on **December 17, 2024** and may be verified on the AJE website using the verification code **21ED-2B3B-4B0F-44A1-6B72**.

Neither the research content nor the authors' intentions were altered in any way during the editing process. Documents receiving this certification should be English-ready for publication; however, the author has the ability to accept or reject our suggestions and changes. To verify the final AJE edited version, please visit our verification page at aje.com/certificate. If you have any questions or concerns about this edited document, please contact AJE at support@aje.com.

AJE provides a range of editing, translation, and manuscript services for researchers and publishers around the world. For more information about our company, services, and partner discounts, please visit aje.com.
