# Supplementary material for: Ferroptosis boosting system based on a sonodynamic therapy cascade-augmented strategy for triple-negative breast cancer therapy
Source: Regen Biomater. 2025 May 20;12:rbaf042. doi: 10.1093/rb/rbaf042 (PMC12188201; doi:10.1093/rb/rbaf042)
Supplement: rbaf042_Supplementary_Data [file rbaf042_supplementary_data.zip › Supporting information.docx]

**Supporting information**

# Ferroptosis boosting system based on a sonodynamic therapy cascade-augmented strategy for triple-negative breast cancer therapy

Ju-Ying Zhang^a,b,1^, Han-Mei Li^a,b,1^, Li-Tao Ye^a,b^, Yi-Han Leng^a^, Xiao-Qing Wang^a^, You Yang^a^, Qiong Jiang^a^, Lin-Li Feng^a^, Ling Li^a^, Yang Li^a,*^, and Jin-hong Yu^a,*^

^a^ School of Medical Imaging, North Sichuan Medical College, Nanchong, 637000 Sichuan, China

^b^ Innovation Centre for Science and Technology of North Sichuan Medical College, Nanchong, 637000 Sichuan, China

* Corresponding author.

E-mail addresses: yujinhong@nsmc.edu.cn (Jin-hong Yu), 552410618@qq.com (Yang Li).

^1^ These authors contributed equally to this work.

**Materials and methods**

**Materials**

N-Hydroxy succinimide (NHS), 1-ethyl-3-(3-dimethylaminopropyl) carbodiimide (EDC), methylene blue trihydrate (MB), bovine serum albumin (BSA) and 1,3-diphenylisobenzofuran (DPBF) were purchased from Aladdin Biochemical Technology Co., Ltd. (Shanghai, China). Chlorine e6 (Ce6) was purchased from Shanghai Macklin Biochemical Technology Co., Ltd. (Shanghai, China). 2,7-Dichlorofluorescein diacetate (DCFH-DA) was purchased from Aldrich Chemical Co. (St. Louis, MO, USA). The Calcein-AM/PI and CCK-8 assays were purchased from Beyotime Biotechnology Co., Ltd. (Shanghai, China). RPMI 1640, penicillin/streptomycin and FBS were purchased from Gibco (Thermo Fisher, USA). 4′,6-Diamidino-2-phenylindole (DAPI) was purchased from Solarbio Science & Technology Co., Ltd. (Beijing, China). A mitochondrial membrane potential assay kit (with JC-1) was purchased from Elabscience & Technology Co., Ltd. (Wuhan, China).

**Characterization of CMB NPs**

The hydrodynamic diameter and zeta potential of the CMB NPs were determined using Dynamic Light Scattering (DLS) (Zetersizer Nano ZS, Malvern, UK). The morphology of the CMB NPs was observed via transmission electron microscopy (TEM) (Hitachi, HT7700, Japan). The stability of CMB NPs in PBS containing 10% serum was assessed over a period of 24 h, and the hydrodynamic size index was determined. The elemental composition and valence state of the CMB NPs were evaluated by X-ray photoelectron spectroscopy (XPS) (K-ALPHA, Thermofisher, America) and element mapping. The successful loading of Ce6 on the CMB NPs was analyzed by UV−Vis spectrophotometry (UV-2600i) and Fourier-transform infrared (FTIR) spectra. The encapsulation efficiency (EE) of Ce6 on the CMB NPs was determined via the dialysis method.

**Cell cultures and animals**

4T1 cells were cultured in RPMI 1640 medium supplemented with 10% FBS and 1% penicillin/streptomycin at 37 °C under 5% CO_2_. Exponentially growing cells were used in all experiments.

Female BALB/c normal mice (6–8 weeks, 18–20 g) were purchased from the Animal Center, North Sichuan Medical College. All animal experiments were approved by the Animal Research Committee of North Sichuan Medical College and performed following the Guide for the Use and Care of Laboratory Animals. The study protocol was reviewed and approved under the protocol number (2024001).

**Cellular uptake**

To assess the uptake of CMB NPs by cancer cells, 4T1 cells were seeded in 6-well plates (1 × 10^6^ cells per well) and cultured at 37 °C for 24 h. Afterward, the cells were incubated with CMB NPs for 2, 4, 6, or 8 h. Subsequently, the cells were washed three times with PBS to remove extracellular CMB NPs, detached with trypsin, and resuspended in PBS (0.5 mL). The proportion of 4T1 cells associated with CMB NPs was analyzed using a flow cytometer (SONY SA-3800) with excitation/emission wavelengths of 633/660 nm.

**In vitro cytotoxicity**

4T1 cells were seeded in a 96-well plate at a density of 1×10^4^ cells per well (under humidified 5% carbon dioxide at 37 °C) overnight to allow the attachment of the cells. The next day, the medium in each well was replaced with 100 µL of complete medium containing CMB NPs at different concentrations (0, 3, 6, 9, 12, 15, 18 or 21 µg/mL), and 0.1 mM H_2_O_2_ was added to each well to imitate the tumor microenvironment; the samples were then cocultured for 20 h. Next, the irradiation groups were irradiated by ultrasound for 5 min and then cultivated for 4 h. Subsequently, 100 μL of 1640 (without FBS) medium, which contained 10 μL of standard CCK-8 solution, was added to each well and incubated for another hour. The absorbance of the CCK-8 solution was measured at 450 nm by a microplate reader.

For observation of live and dead cells via CLSM, 4T1 cells were initially costained with propidium iodide (PI, stain for dead cells) and calcein AM (AM, stain for live cells), after which images of different groups (control, US, Ce6-BSA, Ce6-BSA + US, CMB, CMB + US, each group with 0.1 mM H_2_O_2_) were obtained, and the intensities of red fluorescence and green fluorescence were recorded. In addition, the apoptosis rates of the above-treated 4T1 cancer cells were also measured via flow cytometry. The US irradiation parameters were set to 1 MHz, 50% duty cycle, 2 W/cm^2^, and 2 min.

**Validation of intracellular O_2_**

Ru(dpp)_3_Cl_2_ is a fluorescence probe used to determine the intracellular evolution of O_2_. Six-well plates were used to seed and culture 4T1 cells for 24 h. After staining with 5 μM Ru(dpp)_3_Cl_2_ for 4 h, the 4T1 cells were then incubated with Ce6-BSA or CMB NPs,each group with 0.1 mM H_2_O_2_. The fluorescence intensity of Ru(dpp)_3_Cl_2_ was obtained with a flow cytometer.

**Intracellular ROS generation**

4T1 cells were seeded in a 12-well plate at a density of 1×10^5^ cells/well overnight, subsequently, H_2_O_2_ was added, and the incubation was continued for 4 hours. The cells were then treated with Ce6-BSA or CMB NPs for 10 h. The cells were washed with PBS and incubated with 5 μM 2’7’-DCFH-DA in the dark for 30 min. The cells were irradiated with or without US (1 MHz, 2 W/cm^2^, and a 50% duty cycle) for 2 min. After irradiation, the cells were washed with PBS three times, and the level of intracellular ROS generation was determined by flow cytometry (SONY SA-3800) and an Olympus microscope.

**Evaluation of the changes in the mitochondrial membrane potential**

4T1 cancer cells were cultured on a confocal dish for 24 h. Next, the medium was replaced with fresh medium for the various formulations (control, US, Ce6-BSA, Ce6-BSA + US, CMB, CMB + US, each group with 0.1 mM H_2_O_2_). After 12 h of incubation, the tumor mitochondria were stained with the cationic dye JC-1 and then visualized with an Olympus fluorescence microscope.

**Cellular lipid peroxidation evaluation**

The levels of LPO in 4T1 cells treated under different conditions were examined following the guidelines outlined in the C11-BODIPY 581/591 and MDA assay kit manuals.

**Acridine orange (AO) staining**

4T1 cells were cultured in glass dishes at a density of 10,000 cells/well for 12 h. Following different treatments, the cells were washed with PBS three times and then stained with AO dye solution (2 μg/mL, 1 mL) for 15 min. Next, the cells were washed with PBS and examined using an Olympus fluorescence microscope.

**Measurement of intracellular GSH consumption**

4T1 cells (approximately 5 × 10^5^ per well) were plated in 6-well culture dishes and incubated overnight at 37 °C. The cells were subsequently incubated with different samples for 12 h, after which the cells were collected by centrifugation. The GSH levels in 4T1 cells subjected to various treatments were measured with GSH and GSSG assay kits.

**Biotransmission electron microscopy (bio-TEM) assay**

Bio-TEM is widely recognized as the most reliable method for visualizing the morphology of intracellular mitochondria and lysosomes. Following a 24-h incubation with the CMB NPs, the cells were rinsed with PBS and collected using a cell scraper. The harvested cells were fixed with 2.5% glutaraldehyde and processed into ultrathin sections. Finally, the sections were examined using bio-TEM.

**Western blotting**

After being treated with different formulations, the collected cells were lysed via vigorous sonication in an ice bath using ice-cold RIPA lysis buffer, and the corresponding protein concentration was determined with a BCA kit. The protein lysates were then separated using 10–12% SDS‒PAGE and transferred to a polyvinylidene fluoride (PVDF) membrane. The transferred membranes were blocked with 5% skim milk solution for 1 h. Thereafter, immunoblotting of the cell lysates was performed using antibodies against SLC7A11 (1:1000; Huabio), GPX4 (1:2000; Huabio), FTH (1:5000; Huabio), p62 (1:10000; Huabio) and LC3B (1:2000; Huabio) at 4 °C, according to standard protocols. The next day, after being washed with TBST solution, the membranes were reincubated with the corresponding secondary antibody for 1 h at 25 °C. Finally, chemiluminescence reagents were used for visualization, and ImageJ software was used for quantitative analysis.

**Fe^2+^ level test**

4T1 cells were seeded in 10 cm plates and then subjected to different treatments for 24 h. After washing three times with PBS, the cell debris pellets were collected. Next, an Iron Assay Kit was used to determine the Fe^2+^ concentration. After incubation for 10 min at 37 °C, the absorbance was detected with a microplate reader at a wavelength of 593 nm.

**In vivo anti-cancer performance and mechanism analysis**

To establish a tumor model, female BALB/c mice (5 weeks) were subcutaneously inoculated with 4T1 cells (≈1 × 10^6^ cells) in the right flank (primary tumor). Once the tumors reached approximately 100 mm^3^ in size, the tumor-bearing mice were randomly divided into six treatment groups: the control, US, Ce6-BSA, Ce6-BSA + US, CMB, and CMB + US groups. The control group received no treatment. The Ce6-BSA and Ce6-BSA+US groups received intratumoral Ce6-BSA (10 mg/kg) on Days 0, 2, 4, 6, 8, 10, and 12. The CMB and CMB + US groups received intratumoral CMB NPs (10 mg/kg) on Days 0, 2, 4, 6, 8, 10, and 12. Ultrasound irradiation (1 MHz, 2 W/cm^2^, 50% duty cycle, 2 min) was used for the US, Ce6-BSA + US and CMB + US groups. Throughout the study period, the tumor volume of the mice was regularly monitored to assess treatment efficacy. Tumor length (L) and width (W) were measured with calipers at every treatment, and the tumor volume (V, mm^3^) was calculated. After 14 d, all the mice were sacrificed, and the tumors were removed and preserved in a solution containing 4% formaldehyde for subsequent hematoxylin and eosin (H&E) staining. Additionally, tumor slices were subjected to Ki-67 assays, and immunohistochemical staining was used to monitor the levels of LC3 and GPX4 expression in tumor tissues. In addition, after intratumoral injection of 200 μl CMB NPs in tumor-bearing mice, real-time monitoring and image signal acquisition were conducted using ultrasound imaging technology. The ultrasound signal intensity was quantitatively analyzed using the ImageJ software.

**Biosafety evaluations**

First, the body weight of each group was monitored with every treatment as an indicator of systemic toxicity. Second, a hemolysis test was performed: mouse blood (1 mL) was collected and centrifuged (3000 rpm, 3 min); the red blood suspensions were diluted 50 times with PBS and dispensed into 1.5 mL centrifuge tubes of 500 μL each; and distilled water (positive control), PBS (negative control), and different concentrations of CMB NPs (0, 3, 6, 9, 12, 15, 18 or 21 µg/mL) were added to the centrifuge tubes. After incubation at 37 °C for 1 h, the hemolysis rate was measured at 572 nm with a UV−Vis spectrophotometer. Finally, the mice were subjected to different treatments: normal saline (control), US, Ce6-BSA, Ce6-BSA + US, CMB, and CMB + US. After 14 d, the major organs (heart, liver, spleen, lungs, and kidneys) were weighed and collected for hematoxylin and eosin staining.

**Blood drug concentration experiment**

To evaluate the in vivo pharmacokinetics of CMB NPs, subcutaneous injections of CMB NPs (10 mg/kg) were administered, and blood samples were collected at the following time points: 0 min, 30 min, 1 h, 2 h, 4 h, 6 h, 12 h, and 24 h. A 10 µL plasma sample was collected at each time point and diluted with 200 µL acidic methanol. The sample was then centrifuged at 10000 rpm for 10 minutes. The supernatant was analyzed for absorbance at 403 nm using a multifunctional microplate reader.

**Statistical analysis**

All data are presented as the mean ± standard deviation (SD), and the statistical analysis of the data was performed using one-way ANOVAs. The statistical significance level was set as *P* < 0.05.

Supplementary Fig. 1. Size distribution of CMB NPs.

Supplementary Fig. 2. Zeta potential of CMB NPs.

Supplementary Fig. 3. The stability of CMB NPs.

Supplementary Fig. 4. XPS comparative spectra of CMB NPs.

Supplementary Fig. 5. The characteristics of O_2_ production by Ru(dpp)_3_Cl_2_.


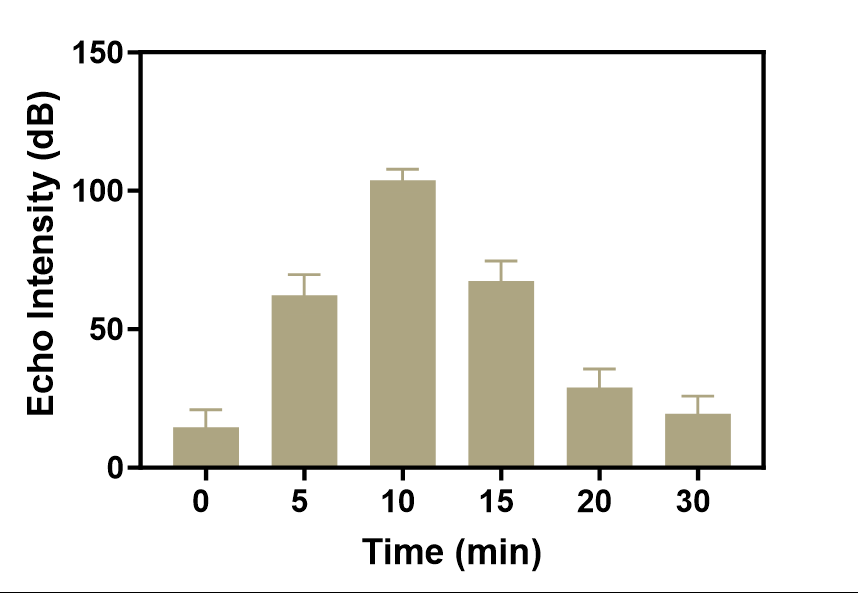


Supplementary Fig. 6. The corresponding echo intensities of CMB NPs in vitro.

Supplementary Fig. 7. The ^1^O_2_ production of CMB NPs.

Supplementary Fig. 8. The safety of CMB NPs.


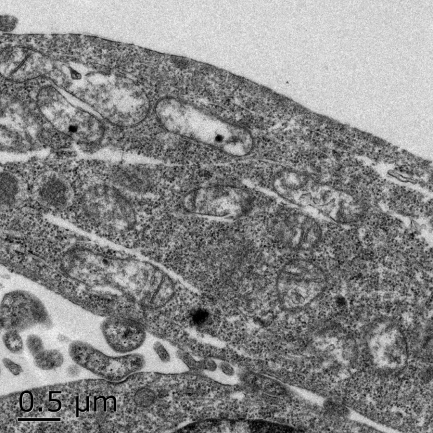


Supplementary Fig. 9. The reduction in mitochondrial ridges (red arrow) in CMB+US group.


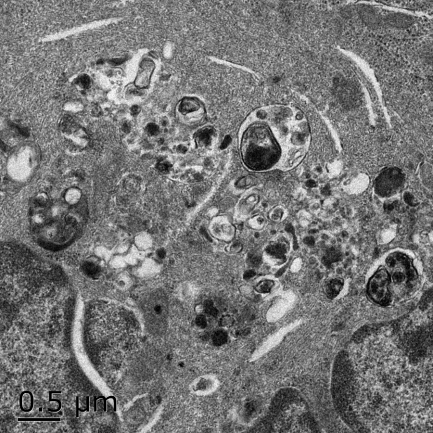


Supplementary Fig. 10. The atypical accumulation of autophagosomes and other autophagic vesicles (red arrow) in CMB+US group.

**B**

**A**

Supplementary Fig. 11. (A) Volume inhibition rate after different treatments. (B) Weight inhibition rate after different treatments. Data are presented as mean ± SD, *****P* < 0.0001.


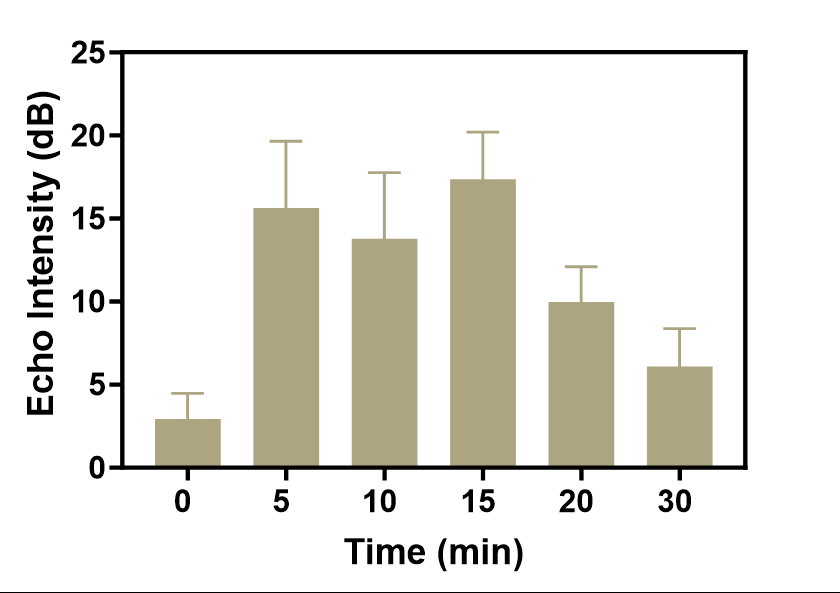


Supplementary Fig. 12. The corresponding quantitative analysis of ultrasound imaging.

Supplementary Fig. 13. Major organs indexes of mice after different treatments. Data are presented as mean ± SD.


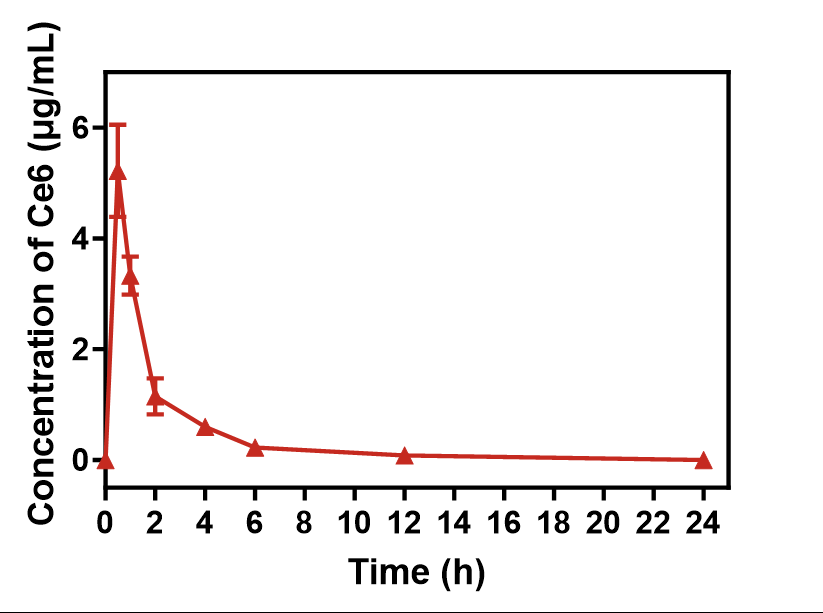


Supplementary Fig. 14. Pharmacokinetics of CMB NPs. Data are presented as mean ± SD.
